# The Serine Protease Domain of MASP-3: Enzymatic Properties and Crystal Structure in Complex with Ecotin

**DOI:** 10.1371/journal.pone.0067962

**Published:** 2013-07-04

**Authors:** Christine Gaboriaud, Rajesh Kumar Gupta, Lydie Martin, Monique Lacroix, Laurence Serre, Florence Teillet, Gérard J. Arlaud, Véronique Rossi, Nicole M. Thielens

**Affiliations:** 1 Institut de Biologie Structurale (IBS), Direction des Sciences du Vivant, Commissariat à l’Energie Atomique et aux Energies Alternatives, Grenoble, France; 2 IBS, Centre National de la Recherche Scientifique, Grenoble, France; 3 IBS, Université Grenoble Alpes, Grenoble, France; Instituto de Tecnologica Química e Biológica, UNL, Portugal

## Abstract

Mannan-binding lectin (MBL), ficolins and collectin-11 are known to associate with three homologous modular proteases, the MBL-Associated Serine Proteases (MASPs). The crystal structures of the catalytic domains of MASP-1 and MASP-2 have been solved, but the structure of the corresponding domain of MASP-3 remains unknown. A link between mutations in the *MASP1/3* gene and the rare autosomal recessive 3MC (Mingarelli, Malpuech, Michels and Carnevale,) syndrome, characterized by various developmental disorders, was discovered recently, revealing an unexpected important role of MASP-3 in early developmental processes. To gain a first insight into the enzymatic and structural properties of MASP-3, a recombinant form of its serine protease (SP) domain was produced and characterized. The amidolytic activity of this domain on fluorescent peptidyl-aminomethylcoumarin substrates was shown to be considerably lower than that of other members of the C1r/C1s/MASP family. The *E. coli* protease inhibitor ecotin bound to the SP domains of MASP-3 and MASP-2, whereas no significant interaction was detected with MASP-1, C1r and C1s. A tetrameric complex comprising an ecotin dimer and two MASP-3 SP domains was isolated and its crystal structure was solved and refined to 3.2 Å. Analysis of the ecotin/MASP-3 interfaces allows a better understanding of the differential reactivity of the C1r/C1s/MASP protease family members towards ecotin, and comparison of the MASP-3 SP domain structure with those of other trypsin-like proteases yields novel hypotheses accounting for its zymogen-like properties *in vitro*.

## Introduction

**Figure 1:**
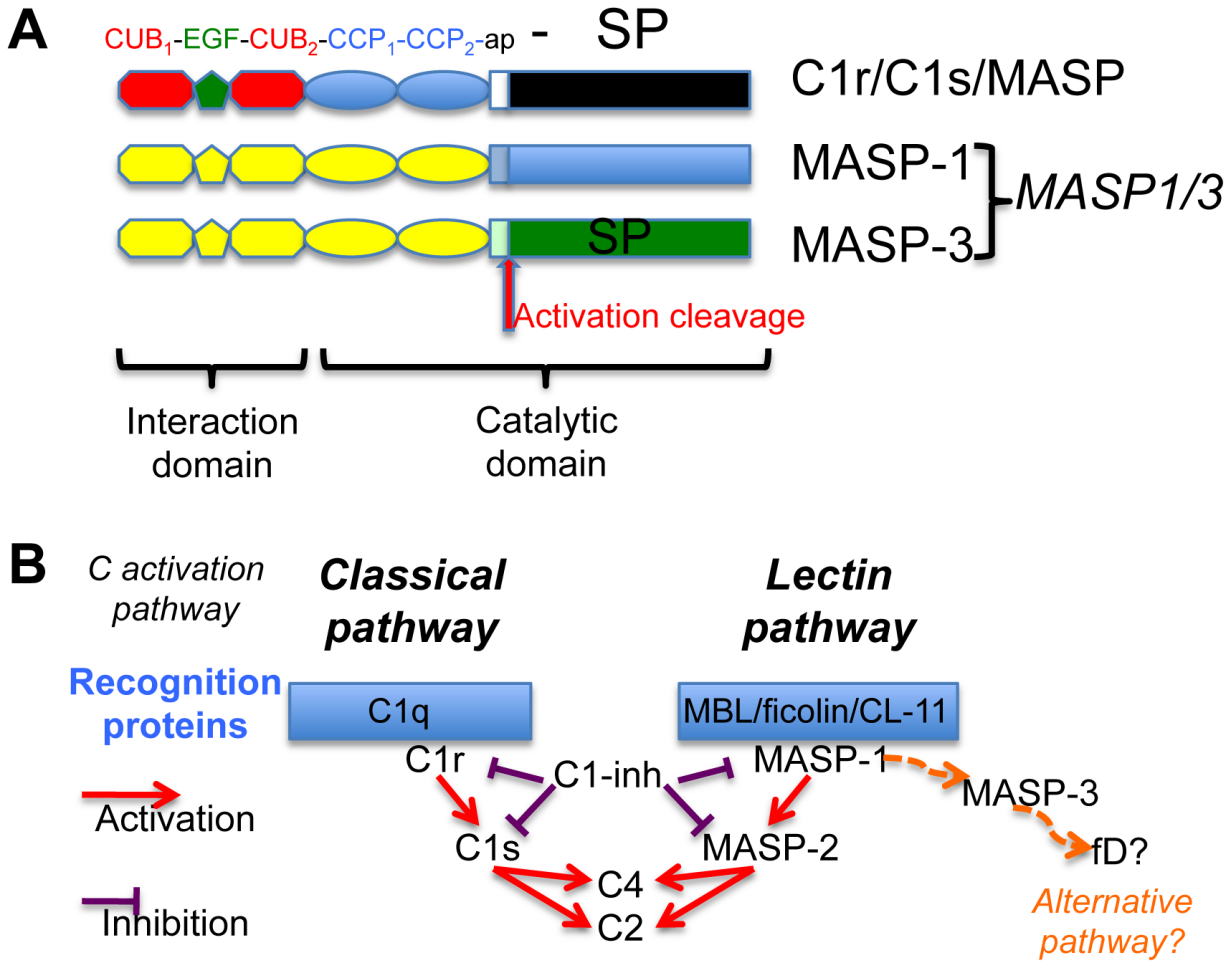
Modular structure of the proteases of the C1r/C1s/MASP family and their role in complement activation. (**A**) The generic modular structure of the proteases of this family is shown. MASP-1 and -3, the two proteases coded by the *MASP1* gene, exhibit a common core (yellow) and unique serine protease (SP) domains. The target of the activation cleavage and the functional domain subdivision are illustrated. The N-terminal interaction domain mediates the binding to the cognate recognition protein and calcium-dependent protease dimerization. In C1r, C1s and MASP-2, the catalytic activity of the C-terminal SP domain is modulated by the preceding CCP1 and CCP2 modules involved in substrate recognition or dimerization [22], [31], [63]. ap: activation peptide. CCP: complement control protein module; CUB: complement C1r/C1s, Uegf, Bmp1 module (**B**) The activation cascades triggering the lectin and classical complement (C) pathways, and their inhibition by C1-inhibitor, are illustrated. The proteases are associated in large complexes with collagen defence recognition proteins. A possible role for MASP-3 in the activation of the complement alternative pathway needs to be confirmed and its other possible implications outside complement are to be deciphered.

Tightly regulated cascades of proteolytic activations control the complement system, a key player of the host humoral defence, as well as essential physiological processes such as coagulation and fibrinolysis. Activation of the classical and lectin pathways of complement is mediated by homologous modular proteases of the C1r/C1s/MASP family (Fig. 1). This process involves large proteolytic complexes including a recognition molecule of the defence collagens family together with its cognate proteases. The recognition proteins of the lectin pathway identified so far encompass mannan-binding lectin (MBL) [1], collectin 11 (CL-11, CL-K1) [2], [3], and ficolins M, L and H (also called ficolin-1, -2 and -3) [4]. Three homologous MBL-associated serine proteases (MASP)-1, -2 and -3, are found associated to these recognition proteins [5], as well as two non-enzymatic components called MAp19 (MBL-associated protein of 19 kDa) or sMAP (small MBL-associated protein) [6], [7] and MAp44 (MBL-associated protein of 44 kDa) or MAP-1 (MBL-associated protein 1) [8], [9]. MAp19 is an alternative splicing product of the *MASP2* gene whereas MASP-1, MASP-3, and MAp44 are all encoded by the *MASP1/3* gene. MASP-1 and MASP-3 only differ by their serine protease domains and the preceding 15 amino acid residues (Fig. 1). All MBL-associated proteins form homodimers able to interact individually with the lectin pathway recognition proteins through their N-terminal interaction domain.

Clear roles have been recently assigned to MASP-1 and -2 in the activation of the complement lectin pathway, paralleling the roles of C1r and C1s in the classical pathway (Fig. 1, [10], [11]). The classical pathway C1 complex comprises a recognition protein C1q and a C1r_2_C1s_2_ tetrameric complex of serine proteases. Binding of C1q to suitable targets triggers self-activation of C1r, which in turn activates C1s, the protease responsible for cleavage of C4 and C2, leading to assembly of the C3 convertase C4b2a [12]. In a similar way, MASP-1 appears to be essential for the activation of MASP-2, the latter cleaving C4 and C2 [10], [11]. MASP-1 can also activate proenzyme C2 in the C4bC2 complex [13].

A regulatory role has been suggested for MASP-3, MAp44 and MAp19, because of their potential ability to compete with MASP-1 and -2 for interaction with the recognition proteins [8], [9], [13], [14], [15]. Initial analysis of recombinant MASP-3 has revealed that it is produced in a proenzyme form, unable to autoactivate [16]. An unexpected important role of the MASP-3 protease in early developmental processes has been recently suggested by the recent discovery of a link between mutations in the *MASP1/3* gene and the 3MC syndrome, a rare autosomal recessive syndrome characterized by various developmental disorders [17], [18]. Three missense mutations were indeed identified in the exon encoding the SP domain of MASP-3 in four independent families affected by this syndrome [17]. In two other independent families, Sirmaci *et al.* [18] identified a nonsense mutation in the part of the gene encoding the interaction region common to MASP-1, MASP-3 and MAp44, and another missense mutation in the SP domain of MASP-3. CL-11 mutations have also been related to the 3MC syndrome, suggesting a possible association between MASP-3 and CL-11 in this physiological context [17]. Other physiological molecular partners of MASP-3 in this context remain to be identified. It has also been proposed that murine MASP-1 and/or MASP-3 (since they are encoded by the same *MASP1* gene) could be involved in activation of the alternative complement pathway through cleavage of pro-factor D [19], but this remains a controversial issue since a recent study showed that in humans both MASPs are dispensable for activation of the alternative pathway [10]. In addition, activation of MASP-3 by MASP-1 has been recently reported [10], [19], [20].

Whereas the crystal structures of the catalytic domains of MASP-1, MASP-2 and of their classical pathway homologues C1r and C1s have all been solved during the past twelve years [21], [22], [23], [24], [25], the structure of the SP domain of MASP-3 remains unknown. To gain deeper insights into the enzymatic and structural properties of MASP-3, several recombinant catalytic fragments including the SP domain were produced, characterized and used for crystallization trials, which proved to be unsuccessful for the free enzyme. The esterolytic activity of these fragments and their interaction with the *E. coli* pan-serine protease inhibitor ecotin were investigated and compared with other proteases, following previous analyses [26]. Ecotin was used as a molecular tool to assist crystallization of the MASP-3 SP domain. The structure of a tetrameric complex, comprising an ecotin dimer and two MASP-3 SP domains, has been solved and refined to 3.2 Å. Although some flexible external loops are not clearly observed, the locations of the four mutations reported to be associated with the 3MC syndrome are clearly defined in the MASP-3 SP structure. By comparison with proteases of the complement and coagulation cascades, new hypotheses accounting for the poor activity of MASP-3 *in vitro* are proposed in light of the structure and of recent findings about possible allosteric mechanisms controlling the activity of several serine proteases [27].

## Materials and Methods

### Materials

Fluorogenic aminomethylcoumarin(AMC)-conjugated substrates were purchased from Peptanova (DPR, FSR, PFR and VPR), Calbiochem (FVR and GGR), American Diagnostica (FGR) and CASLO Laboratory (KGR and APR). AMC was from Sigma. Diisopropylfluorophosphate (DFP) was from Calbiochem. Oligonucleotides were purchased from Eurogentec.

### Proteins

Human plasma thrombin and coagulation factors XIa and XIII were obtained from Calbiochem. Bovine pancreas trypsin (TPCK treated) was from Sigma. Ecotin was purchased from Gentaur Molecular Products and recombinant human insulin-like growth factor-binding protein (IGFBP)-5 from R&D Systems.

C1 inhibitor and activated C1r and C1s were purified from human serum as described by Arlaud *et al.* [28], [29]. Recombinant fragments MASP-1 CCP_1/2_-ap-SP, MASP-2 CCP_1/2_-ap-SP, C1s CCP_2_-ap-SP and C1r CCP_2_-ap-SP, and full-length MASP-3 were produced in baculovirus-infected insect cells and purified as described previously [16], [30], [31], [32]. The C1s fragment CCP_2_-ap-SP was activated by C1r as described previously [31]. The N-terminal extracellular domain of the mature human protease-activated receptor (PAR) 1 was produced in *E. coli* as a GST fusion protein using the pGEX-pP-3/PAR1E plasmid (kindly provided by Dr F. Lanza). The recombinant protein was purified by affinity chromatography on glutathione-Sepharose 4B beads (GE Healthcare) as described by Loew *et al.* [33].

### Production of the Serine Protease Domain of MASP-3

A DNA fragment encoding the activation peptide and the SP domain of human MASP-3 (residues 416–709 of the mature protease) was amplified by PCR using Vent_R_ polymerase (New England Biolabs) and the expression plasmid coding for MASP-3 [16] as a template, according to established procedures. The amplified DNA, containing a BglII restriction site at the 5′ end and a stop codon followed by an EcoRI site at the 3′ end, was cloned in-frame with the melittin signal peptide into the BamHI and EcoRI sites of the pNT-Bac baculovirus transfer vector [31]. This plasmid served as a template to generate the vector coding for the MASP-3 SP domain (residues 431–709) using the QuickChange XL site-directed mutagenesis kit (Stratagene), by deleting the segment coding for the activation peptide and adding two residues (Asp-Leu) at the N terminus, due to in-frame cloning with the signal sequence of melittin. The pNT-Bac-MASP-3 SP plasmid was checked by double-strand sequencing (Genome Express, France). The recombinant baculovirus was generated using the Bac-to-Bac™ system (Invitrogen) and amplified as described previously [34]. High Five insect cells were infected with the recombinant virus for 72 h at 27°C.

The culture supernatant (0.5 L) containing the MASP-3 SP domain was dialyzed against 25 mM NaCl, 50 mM triethanolamine-HCl, pH 7.4, and loaded onto a Q-Sepharose Fast Flow column (2.8×10 cm) (GE Healthcare) equilibrated in the same buffer containing 1 mM iodoacetamide. Elution was conducted by applying a linear gradient to 250 mM NaCl in the same buffer. Fractions containing the recombinant fragment were identified by SDS-PAGE analysis, concentrated by ultrafiltration and final purification was achieved by high-pressure gel permeation on a TSK G3000 SW column (7.5×600 mm) (Tosoh Bioscience) equilibrated in 145 mM NaCl, 50 mM triethanolamine-HCl, pH 7.4. The purified fragment was concentrated to 0.5–1 mg/ml by ultrafiltration and stored at −20°C.

The tetrameric complex was formed by incubating the MASP-3 SP domain and ecotin at a 1∶1 molar ratio at room temperature for 20 min. Fractions containing a maximum amount of 100 *µ*g of MASP-3 SP were injected on the TSK G3000 SW column equilibrated in 145 mM NaCl, 50 mM triethanolamine-HCl, pH 7.4. The first eluted peak, corresponding to the tetrameric complex, was pooled and concentrated to 2–3 mg/ml by ultrafiltration.

### SDS-PAGE Analysis and Chemical Characterization of the Recombinant Proteins

Samples were analyzed by SDS-PAGE followed by Coomassie blue staining of the proteins. N-terminal sequencing (on liquid samples or after SDS-PAGE and electrotransfer) and MALDI mass spectrometry analyses were carried out as described previously [35].

### Amidolytic Assays

The amidolytic activity of MASP-3 SP, thrombin and trypsin on selected peptidyl-AMC substrates was determined using a fluorometric assay based on the measurement of AMC released upon cleavage. Varying enzyme amounts were added to the substrate (0.1 *µ*M) in 2 ml of 20 mM Hepes, 5 mM CaCl_2_, pH 8.5. Samples were excited at 360 nm and emission was read at 440 nm every 30 s for 30 min at 37°C using an Aminco-Bowman Series 2 fluorometer. The initial rates of AMC release were calculated from a calibration curve obtained with varying AMC concentrations (1–10 *µ*M) diluted in 2 ml of the above buffer. Rates were expressed in pmol AMC released/min/*µ*g of enzyme to allow comparison with previously published data.

For selected substrates, the Michaelis constant (*K*_m_) and the maximum velocity (*V*_max_) were determined for MASP-3 SP and thrombin, using nonlinear regression analysis (SigmaPlot software). The substrate concentrations varied from 50 to 200 *µ*M and the enzyme amounts used were 0.01 *µ*g for thrombin and 7.5 *µ*g for MASP-3 SP.

The effect of protease inhibitors was evaluated by measuring the residual activity of MASP-3 SP on the VPR- or FGR-AMC substrates after preincubation of the enzyme with ecotin for 20 min at room temperature, with C1 inhibitor for 1 h at 37°C, and with 5 mM diisopropylfluorophosphate (DFP) for 30 min at 37°C.

### Proteolytic Assays

The proteolytic activity of MASP-3 SP, thrombin and C1s was analyzed by incubation of 2.5–3.5 *µ*g of MASP-3, IGFBP-5, factor XIII and the extracellular domain of PAR1 at 37°C for 4–16 h with MASP-3 SP (0.1–0.2 molar ratio), C1s (0.2 molar ratio) or thrombin (0.1–1% w/w) followed by SDS-PAGE of the incubation mixtures under reducing conditions.

### Surface Plasmon Resonance Spectroscopy and Data Evaluation

Surface plasmon resonance analyses were performed using a BIAcore 3000 or a Biacore X instrument (GE Healthcare). Ecotin (1,000 RU) was immobilized using the amine coupling chemistry by injecting the protein (diluted to 20 *µ*g/ml in 10 mM sodium acetate, pH 5.0) on the surface of a CM5 sensor chip (GE Healthcare) in 150 mM NaCl, 5 mM EDTA, 10 mM HEPES, pH 7.4 containing 0.005% surfactant P20 (GE Healthcare). Binding was measured at a flow rate of 20 µl/min/min in 145 mM NaCl, 50 mM triethanolamine-HCl, pH 7.4 containing 0.005% surfactant P20. Regeneration of the surface was achieved by 10-*µ*l injections of 10 mM HCl. Equivalent volumes of each protein sample were injected over a reference surface without immobilised protein for subtraction of the bulk refractive index background.

Data were analysed by global fitting to a 1∶1 Langmuir binding model of both the association and dissociation phases for at least five concentrations simultaneously using the BIAevaluation 3.2 software (GE Healthcare). The apparent equilibrium dissociation constants (*K*_D_) were calculated from the ratio of the dissociation and association rate constants (*k*_d_/*k*_a_). Although the interaction of the ecotin dimer with proteases is likely more complex than a simple 1∶1 binding model, data fitting using this model yielded satisfactory chi2 values (<4) and was used for comparison purposes.

### Crystallization, Structure Determination, and Refinement

The ecotin/MASP-3 SP complex was concentrated to about 7 mg/ml in a buffer containing 50 mM triethanolamine-HCl pH 7.4, 145 mM NaCl and 100 mM sulfobetain NDSB195. Reproducible crystals were obtained by the vapour diffusion method at 20°C using a reservoir solution containing 18–22% (w/w) PEG2KMME, 0.2 M ammonium sulfate, 0.1 M sodium acetate pH 4.7. PEG 400 (3% w/v) was also often added to improve crystal quality. Very thin plate-like crystals grew in two weeks. Several datasets were collected on the ESRF beamlines ID14-eh1, ID14–eh2, ID29 and BM14. These were integrated and merged using XDS [36]. The crystals belong to the P2_1_2_1_2 space group, with cell dimensions a = 66.7 Å, b = 164.6 Å, c = 90.6 Å. Their diffraction limit is about 3.2 Å, with a high mean Wilson B factor of about 60 Å^2^. Their solvent content is about 54%, with one tetramer per asymmetric unit. The structure was solved by molecular replacement using Phaser [37], defining the two positions of the SP domain and inhibitor search models. The ecotin search model was a mutant version extracted from the Protein Data Bank (PDB) entry 1XXF describing its complex with the factor XIa catalytic domain [38]. For the SP domain, a truncated version of the MASP-2 SP domain was used [25], in which the tips of the more variable loops (463–467, 484–495, 504–510, 526–529, 556–567, 577–581, 604–611, 685–686) were removed. A clear unique solution was obtained. Moreover, several residue substitutions from the models were visible in the initial electron density maps. Numerous runs of iterative model building and refinement were performed, using several graphics and refinement softwares (O [39], Coot [40], CNS [41], BUSTER [42], Refmac [43] to check and improve the accuracy of the model. Bulk solvent correction and Translation Libration Screw-motion (TLS) refinement were used, each chain defining one TLS group. Non-crystallographic symmetry (NCS) restraints were included in the refinement, carefully excluding only the flexible zones for which the two copies display significant different electron density because the molecules are in a slightly distinct environment. An illustration of the quality of the final electron density map together with the corresponding model is provided (Fig. S1). Missing parts in the current model include residues 1–5 and 89 in ecotin, together with the following MASP-3 segments: 456–458, 508–511, 578–593 and 705–709. The coordinates and structure factors of the ecotin/MASP-3 SP complex are accessible (PDB code 4IW4).

## Results and Discussion

### Production and Characterization of the Recombinant SP Domain of MASP-3

**Figure 2:**
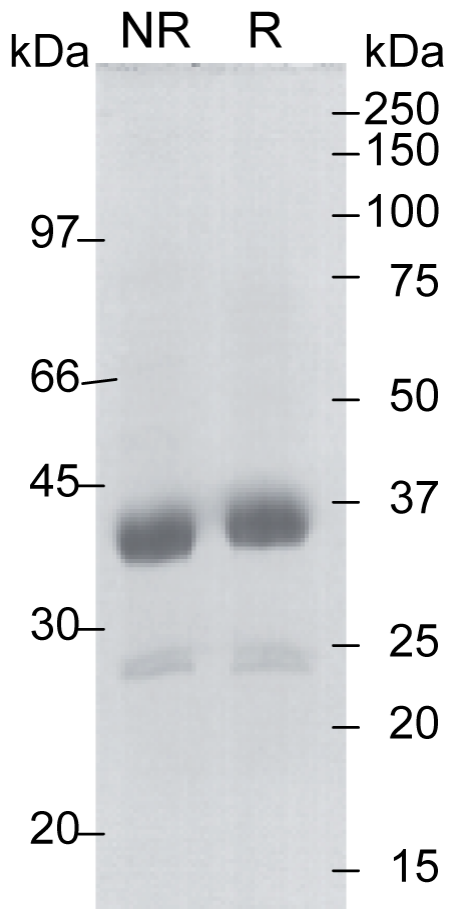
SDS-PAGE analysis of the recombinant MASP-3 SP domain. The purified SP domain of MASP-3 was analyzed by SDS-PAGE under non-reducing (NR) and reducing (R) conditions followed by Coomassie blue staining. The molecular masses of reduced and non-reduced standard proteins (expressed in kDa) are shown on the right and left sides of the gel, respectively.

The main objective of this study was to determine the structure of the catalytic domain of MASP-3. For this purpose, artificial processing of the melittin signal peptide was used to generate a recombinant fragment of the SP domain of MASP-3 starting after the activation cleavage site at Ile^431^. This strategy aimed to obtain a conformational ‘active’ state of the SP domain, as expected after its activation cleavage, overcoming the inability of MASP-3 to undergo self-activation [16]. A similar fragment was previously produced by Cortesio & Jiang [26] using a mammalian cell expression vector containing the signal peptide of human CD33. The recombinant SP domain was produced in baculovirus-infected insect cells and purified from the cell culture supernatant as described under *Materials and Methods*. The purification yield from 500 ml of culture supernatant was 700 *µ*g. SDS-PAGE analysis of the purified fragment yielded a band migrating at about 35 kDa under both non-reducing and reducing conditions, corresponding to the expected mass (Fig. 2). In some preparations, a doublet of lower mass (25 kDa) was detected, accounting for about 5% of the purified material, and corresponding to a degradation product. N-terminal sequence analysis yielded a major sequence, Ile^431^-Ile-Gly-Gly-Arg-Asn-Ala-Glu…, corresponding to that expected for the SP domain, indicating that the melittin signal peptide had been correctly processed. A minor sequence, Gly^433^-Gly-Arg-Asn-Ala-Glu-Pro-Gly…, accounting for about 20% of the recombinant material, was also obtained, corresponding to a fragment lacking the first two N-terminal residues. Mass spectrometry analysis yielded a heterogeneous peak centered on a mass value of 34,300±27 Da. Given the predicted mass of the polypeptide (30,551 Da), the deduced mass value for the carbohydrate moieties (3,749 Da) is consistent with the presence of three *N*-linked oligosaccharide chains comprising two *N*-acetylglucosamine and five mannose residues (calculated mass 1,218 Da). These data indicate that all predicted *N*-glycosylation sites [14] are occupied by short high-mannose oligosaccharides, in accordance with previous data on full-length recombinant MASP-3 expressed in the same system [16].

### Enzymatic Activity of the MASP-3 SP Domain

The catalytic activity of the recombinant SP domain of MASP-3 and its inhibition pattern were checked and further investigated. Among the nine peptidyl-AMC substrates listed in Table 1, the highest enzymatic activity was observed with substrates of tissue plasminogen activator (FGR), kallikrein (PFR) and thrombin (VPR). Significant cleavage was also observed for DPR, FSR, GGR and KGR, which are substrates for thrombin/trypsin, factor XIa, thrombin, and trypsin, respectively. We observed very low cleavage of the thrombin substrate FVR-AMC and no cleavage of APR-AMC, which mimics the cleavage site of PAR4, a protein substrate of MASP-1 [44]. The values obtained for cleavage of PFR, VPR and GGR and for the control enzyme thrombin are in the same range as those obtained previously [26], [44], [45]. Nevertheless, the amidolytic activity of MASP-3 SP is considerably lower than that of thrombin, trypsin or other members of the C1r/C1s/MASP family (Table 1, [45]). For example, the initial rate of AMC release (in pmol/min/*µ*g enzyme) for its best substrate FGR is about 28 in MASP-3, which is very low compared to 80 for MASP-2, 750 for C1s, 2700 for thrombin and 16800 for MASP-1. Determination of the kinetic parameters for the FGR and VPR substrates yielded *K*_m_ values of 410 and 243 *µ*M, respectively, consistent with that obtained previously for MASP-3 SP and about 10 times lower than those determined for thrombin (Table 2). The difference in the *V*_max_ values was even more important (40- to 400-fold) (Table 2), confirming the very low peptide cleaving efficiency of MASP-3 SP. As observed previously for full-length MASP-3, the FGR- and VPR-AMC cleavage activity was completely blocked by pretreatment of the protease with 5 mM DFP, whereas preincubation with C1-inhibitor at inhibitor:protease ratios up to 4∶1 did not affect the amidolytic activity of MASP-3. Incubation of the MASP-3 SP domain with the bacterial protease inhibitor ecotin at a 1∶1 molar ratio resulted in 82% inhibition of FGR-AMC cleavage, reflecting the efficiency of this inhibitor, in agreement with previously published data [26].

**Table 1:** Amidolytic activity of MASP-3 SP, thrombin and trypsin.

Substrate (known for)	MASP-3 SP	thrombin	trypsin
FGR-AMC (tPA)	18.2	1990 (2700^a^)	
PFR-AMC (plasmin, kallikreins)	16.1 (11.0^b^)	23	
VPR-AMC (thrombin, factor XIII, kallikreins)	12.8 (9^b^)	14720 (15710^a^)	
DPR-AMC (thrombin, trypsin)	6.7	92430	
FSR-AMC (factor XIa)	4.4	450 (250^a^)	
GGR-AMC (tPA, urokinase, thrombin)	4.1 (5.6^b^)	230 (260^a^)	
KGR-AMC (trypsin)	3.8	246	20170
FVR-AMC (thrombin)	0.93	983	
APR-AMC (trypsin)	0	1290	12930

Activity is expressed as initial rates of AMC released (pmol/min/*µ*g enzyme).

The values determined in the present study are indicated. Alternative values from ^a^Presanis et al. [45] and ^b^Cortesio and Jiang [26] are given as additional references within brackets.

**Table 2:** Kinetic parameters for selected substrates of MASP-3 SP and thrombin.

	FGR-AMC		VPR-AMC	
enzyme	*K*_m_ (*µ*M)	Vmax^a^	*K*_m_ (*µ*M)	Vmax^a^
MASP-3 SP	410	89	243 (250^b^)	45
thrombin	25^c^	3340^c^	27 (9.6^c^)	18360 (32051^c^)

a Vmax is expressed in pmol AMC/min/*µ*g enzyme.

b Value from Cortesio and Jiang [26].

c Values from Tsiftsoglou and Sim [64].

We also examined the capacity of the MASP-3 SP domain to cleave potential protein substrates. No proteolysis of proenzyme MASP-3 could be detected, as previously observed with the extrinsically activated full-length protease [16], thus confirming that MASP-3 is not able to self-activate. The SP domain of MASP-3 did not either cleave the thrombin protein substrates PAR-1, factor XIII and fibrinogen at an enzyme:substrate molar ratio of 1∶10, in contrast to thrombin which cleaved these proteins efficiently (data not shown).

It has been reported previously that the SP domain of MASP-3 cleaves insulin-like growth factor-binding protein 5 [26]. Incubation of IGFBP-5 in the presence of 20% enzyme (mol/mol) overnight at 37°C resulted in almost complete cleavage, although the amount of residual uncleaved protein was difficult to estimate due to its migration at the same position as the MASP-3 SP fragment. As a control, incubation with activated C1s resulted in IGFBP-5 cleavage, but the digestion was more partial (Fig. S2). However, it should be kept in mind that, in addition to C1s and MASP-3, several proteases have been shown to cleave IGFBP-5, among which plasmin, thrombin, elastase, cathepsin G, disintegrin and metalloproteinase domain-containing proteins (ADAM)-9 and 12s, and pregnancy associated plasma proteases A and A2 [46].

The overall conclusion of these experiments is that the recombinant MASP-3 SP domain exhibits poor enzymatic properties *in vitro,* at least towards standard protease substrates. This feature will be further discussed in light of the crystal structure.

### Comparative Analysis of Protease/ecotin Binding

**Figure 3:**
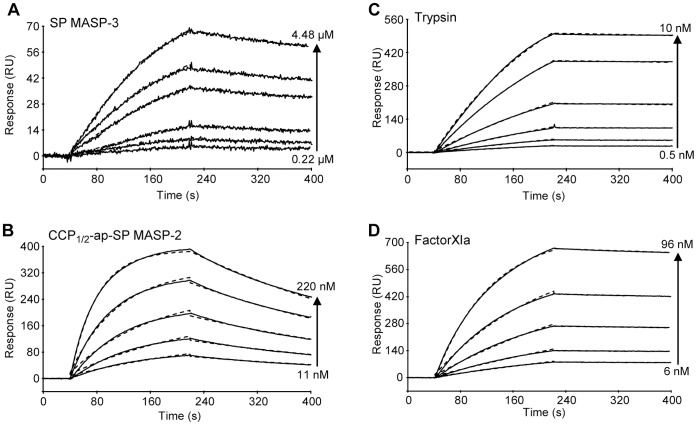
Comparative SPR analysis of the interaction of MASP-3 and other trypsin-like proteases with ecotin. Ecotin (1,000 RU) was immobilized on a CM5 sensor chip as described under *Materials and Methods*. Sixty microliters of varying concentrations of the MASP-3 SP domain (**A**), the MASP-2 CCP_1/2_-ap-SP fragment (**B**), trypsin (**C**), and factor XIa (**D**) were injected over immobilized ecotin in 145 mM NaCl, 50 mM triethanolamine-HCl, pH 7.4 containing 0.005% surfactant P20 at a flow rate of 20 µl/min. The specific binding signals shown were obtained by subtracting the background signal over a reference surface with no protein immobilized. Fits are shown as dotted lines and were obtained by global fitting of the data using a 1∶1 Langmuir binding model.

The fact that the low amidolytic activity of MASP-3 SP domain was significantly inhibited by ecotin prompted us to further investigate its interaction with this inhibitor and compare it with other serine proteases, since ecotin is known as a pan-serine protease inhibitor. Surface plasmon resonance spectroscopy was used to analyze the interaction of different proteases with ecotin. As shown in Fig. 3, the SP domain of MASP-3 readily bound to immobilized ecotin. Kinetic analysis of the binding data at different concentrations (0.22–4.48 *µ*M) yielded association and dissociation rate constants of 1.34×10^3^ M^−1^.s^−1^ and 8.06×10^−4^ s^−1^, respectively, and a resulting apparent equilibrium dissociation constant (*K*_D_) of 601 nM (Table 3). These ecotin binding properties were compared to those of the catalytic domains of MASP-1, MASP-2, C1r, and C1s, as well as of trypsin and factor XI. Whereas no interaction was detected for the C1r and C1s CCP_2_-ap-SP and MASP-1 CCP_1/2_-ap-SP fragments at concentrations up to 2 *µ*M (not shown), trypsin, factor XIa, and the CCP_1/2_-ap-SP fragment of MASP-2 all interacted strongly with immobilized ecotin (Fig. 3). The affinity measured for bovine trypsin is comparable to that determined previously from enzyme inhibition data [47]. The apparent *K*_D_ values determined for MASP-2 CCP_1/2_-ap-SP (24.7 nM), trypsin (0.21 nM) and factor XIa (1.38 nM) are by far lower than that determined for the MASP-3 SP domain (Table 3), indicative of stronger affinities for these three enzymes. The decrease in *K*_D_ values was mainly due to higher association rates (from 78-fold for the MASP-2 fragment to 380-fold for trypsin), combined with moderately lower dissociation rates in the case of trypsin and factor XIa. The possible structural bases for the absence of ecotin binding to MASP-1, C1r and C1s, as well as for the increased association rate observed in the case of MASP-3 will be discussed later.

**Table 3:** Kinetic and dissociation constants for the interaction of selected proteases with immobilized ecotin.

Protease	*k*_a_ (M^−1^ s^−1^)	*k*_d_ (s^−1^)	*K*_D_ (nM)
MASP-3 SP	1.34×10^3^	8.06×10^−4^	601
MASP-2 CCP_1/2_-ap-SP	1.04×10^5^	2.28×10^−3^	24.7
Trypsin	5.09×10^5^	1.09×10^−4^	0.21
Factor XIa	1.23×10^5^	1.69×10^−4^	1.38

### Towards the X-ray Structure of the ecotin/MASP-3 Tetrameric Complex

Interestingly, the low dissociation rate of ecotin with MASP-3 SP domain suggested that their complex could be isolated. This was analyzed by gel filtration, after incubation of the protease with ecotin at various molar ratios. A major objective was indeed to obtain complexes suitable for crystallographic studies, since initial attempts to obtain good crystals of different MASP-3 fragments had failed. Ecotin has been previously described as a molecular tool to assist the crystallization of proteases [38], since the X-ray structures of complexes between ecotin (or variants) and various proteases have been reported, including trypsin [48], collagenase [49], thrombin [50], granzyme B [51], coagulation factors Xa [52] and XIa [38].

**Figure 4:**
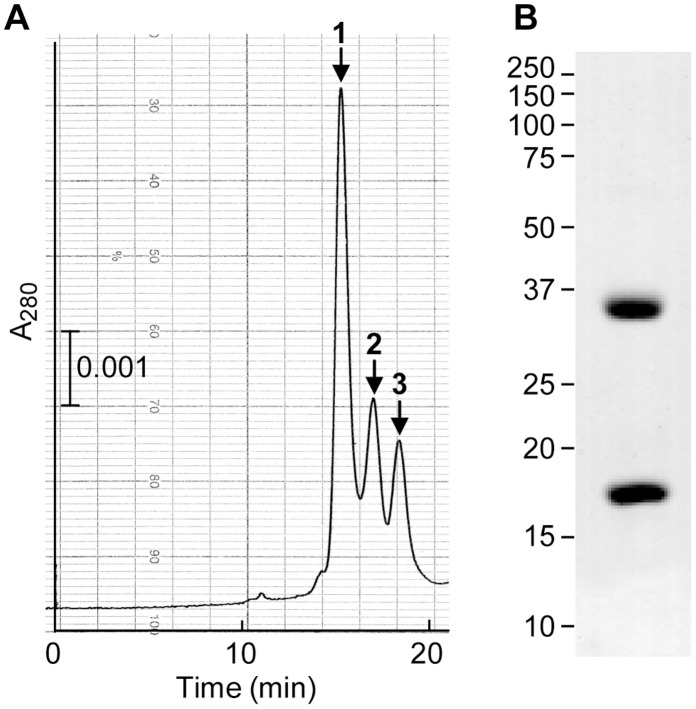
Gel filtration analysis of ecotin-MASP-3 SP complexes. (**A**) Equimolar amounts of the MASP-3 SP domain and ecotin (225 pmol) were preincubated for 20 min at room temperature before injection on a TSK G-3000 SW column equilibrated in 145 mM NaCl, 50 mM triethanolamine-HCl, pH 7.4 and run at 1 ml/min. (**B**) SDS-PAGE analysis under reducing conditions and Coomassie blue staining of the protein content of peak 1. The molecular masses of reduced standard proteins (expressed in kDa) are shown on the left side of the gel.

Three peaks were obtained by gel filtration when using equimolar amounts of protease and ecotin, as shown in Fig. 4A. The major peak 1 contained equal amounts of enzyme and inhibitor, as judged by N-terminal sequencing and SDS-PAGE analysis (Fig. 4B), and corresponded to the tetrameric complex submitted to crystallization. The intensity of the second peak increased when the inhibitor was in excess and was identified as a trimeric complex between the ecotin dimer and the protease, as previously observed for rat trypsin [53]. The third peak corresponded to the uncomplexed protease and the ecotin dimer (molecular mass 32 kDa) and its intensity increased when the protease was in excess.

**Figure 5:**
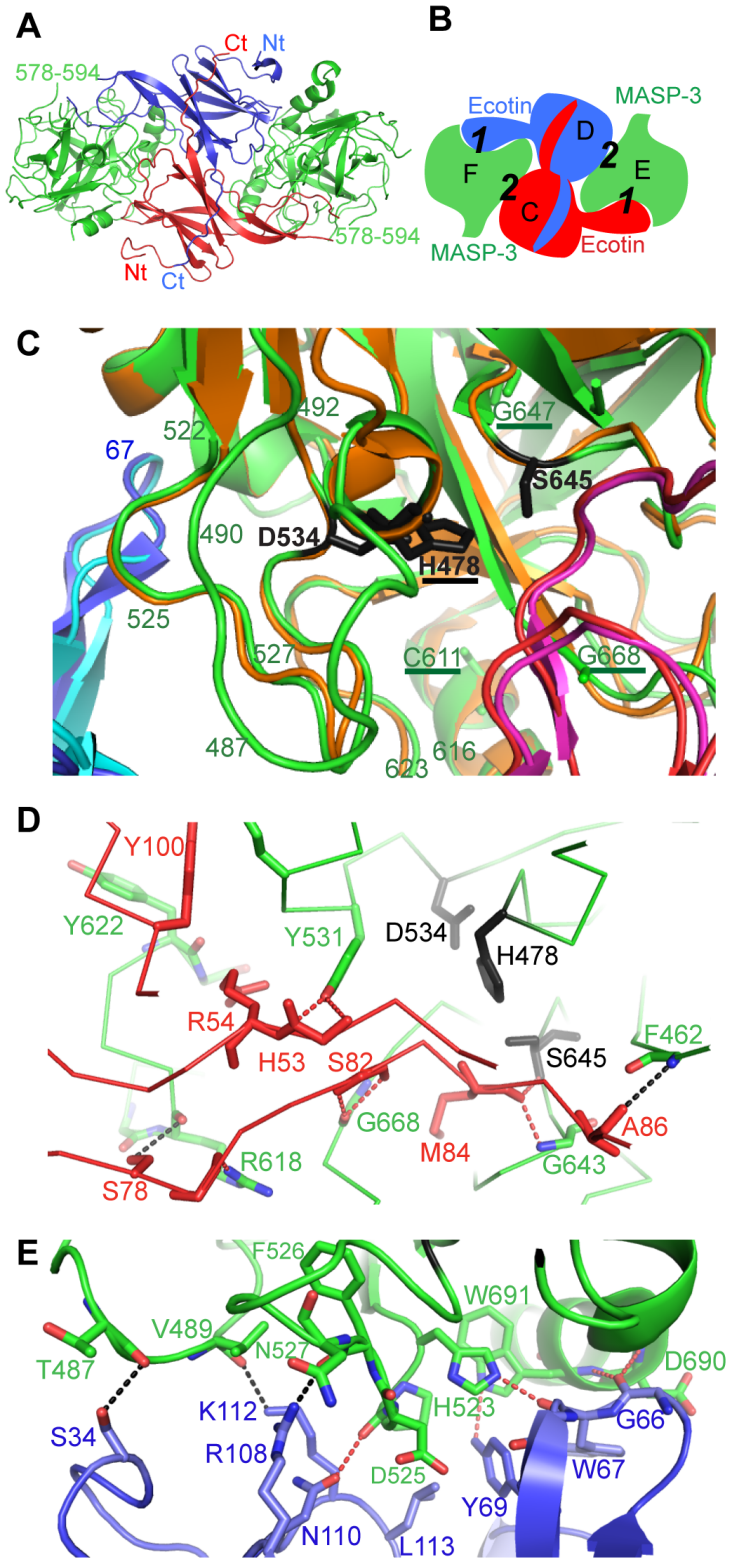
Structure of the ecotin/MASP-3 SP tetramer. (**A**) Overall and (**B**) schematic view of the tetramer highlighting its primary (***1***) and secondary (***2***) interfaces. The same color codes are used for MASP-3 and ecotin in the remainder of the figure. The two ecotin and MASP-3 monomers are labeled C, D and E, F, respectively. (**C**) Zoom showing the MASP-3 active site, clamped in-between two ecotin molecules. The positions of the mutations related to the 3MC syndrome are underlined. The active site triad is in black. The similar tetrameric structure of the Y69F/D70P ecotin mutant complexed to the D102N rat anionic trypsin (PDB code 1EZU) is superimposed, with trypsin colored in orange and the two ecotin chains colored in pink and light blue. Note the large insertion loop B (480–493) in MASP-3 (green) compared to trypsin. (**D**) Zoom on the ecotin/MASP-3 primary interface. For clarity purposes, only the catalytic triad and the residues or main-chain stretches contributing to the closest contacts are shown. (**E**) Zoom on the ecotin/MASP-3 secondary interface. Dotted lines in panels D and E indicate conserved (red) or specific (dark) H-bonds. Here the term ‘conserved’ means that these bonds are observed in the other ecotin-protease complexes.

Crystallization of the ecotin/MASP-3 SP domain tetramer was successful and reproducible. Four coherent datasets were merged to yield a more complete and redundant dataset which was used to solve the structure and refine it to 3.2 Å resolution, with R and Rfree values of 0.208 and 0.269 (Table 4). As observed previously for other ecotin/protease complexes, the tetramer features a central ecotin dimer, each ecotin molecule interacting with both SP domains, hence defining two interfaces (Fig. 5A, B). The primary interface (labeled ***1*** in Fig. 5B) is the one where ecotin directly interacts with the SP domain catalytic triad and substrate-binding site (Fig. 5C, D). Remarkably, the amino acids corresponding to the mutations associated to the 3MC syndrome all cluster in this area (Fig. 5C). The secondary interface (labeled ***2*** in Fig. 5B) corresponds to the interaction with the other ecotin molecule. Two SP surface loops (523–534 and 480–490) are clamped in-between the two ecotin molecules, thus taking part in both interfaces.

**Table 4:** Crystallographic data and refinement statistics.

**Data collection**	
Space group	P2_1_2_1_2
Unit cell lengths (Ål)	a = 66.7, b = 164.6, c = 90.6
Resolution (Ål)	20–3.2
^Rmergea^	11.5 (58.7) b
% completeness^a^	97.1 (90.4) b
I/sigma (I) average^a^	20.13 (4.14) b
No. of unique reflections^a^	16636 (685) b
Redundancy^a^	15.5 (16.1) b
**Refinement**	
Resolution (Ål)	20–3.2
^R^_work_	0.208 (0.259) c
^R^_free_	0.269 (0.349) c
Root mean square deviation χ2 bonds (Å)	0.006
Root mean square deviation χ2 angles (°)	1.16
Residues in Ramachandran favoured region d	670 (91.9%)
Residues in Ramachandran allowed region d	39 (5.3%)
Residues in Ramachandran outlier region d	20 (2.7%)

a Statistics on a merged dataset resulting from 4 crystals collected at ID14-eh2, ID29, ID14-eh1, BM14, ESRF, Grenoble.

b Statistics for the high-resolution bin (3.25–3.20 Å) are in parentheses.

c Statistics for the high-resolution bin (3.28–3.20 Å) are in parentheses.

d Including prolines and glycines – Statistics provided by RAMPAGE [65].

The tetramer is almost perfectly symmetrical, except for slight differences at the edges of the interfaces and for some local differences in the crystal contacts of the two copies of each protein. Compared to other ecotin/protease complex structures, the most similar tetramer is the one formed by the D102N rat anionic trypsin and Y69F/D70P ecotin ([54], PDB code 1EZU). The conformations observed at the primary and secondary interfaces are strikingly conserved in both cases (Fig. 5C, D, E). The global r.m.s.d value between these two tetrameric structures is indeed only 0.95 Å (for 678 aligned residues), and 0.83 Å for the SP domain alone (for 202 aligned residues, with 39.6% sequence identity). In contrast, the MASP-3 480–490 loop features a large insertion compared to trypsin (Fig. 5C).

### Structural Comparison with Homologous Trypsin-like SP Domains

**Figure 6:**
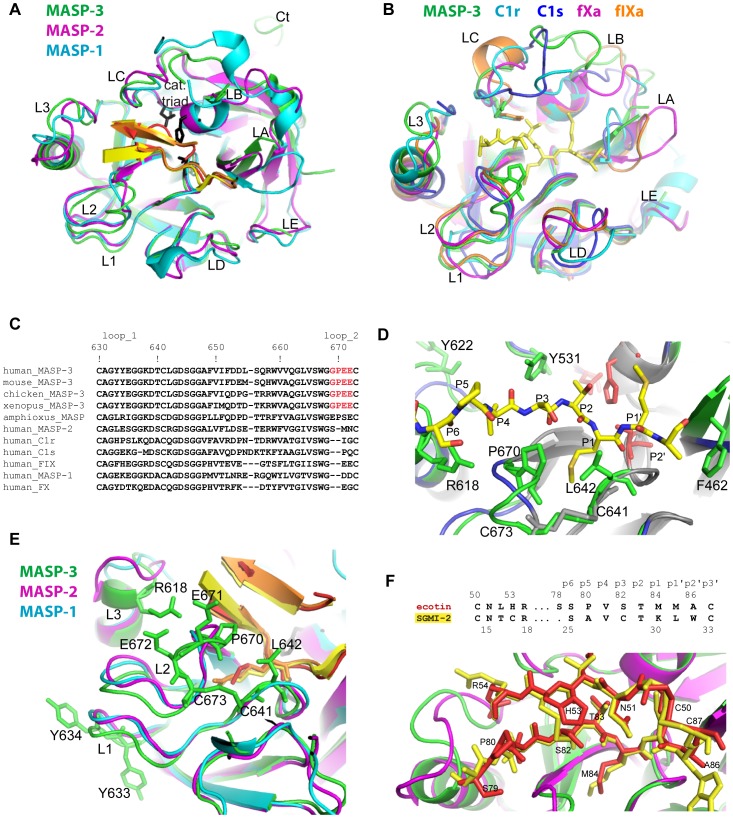
Structural comparison of MASP-3 with other trypsin-like SP domains. (**A**) and (**B**) overall view of the superimposition of the MASP-3 SP domain with different complement and coagulation proteases. The proteases are identified by labels with the corresponding color. The substrate-like ecotin loop 80–86 is displayed in yellow in (**B**). The two similar inhibitor loops are displayed in red (ecotin), orange (SGMI-1) and yellow (SGMI-2) in (**A**). The loop definition is according to [56]: 1: 633–638 (*184–188*); 2: 669–679 (*217–225*); 3: 612–624 (*169–176*); A: 448–463 (*33–42*); B: 480–498 (*59–64*); C: 529–533 (*97–101*); D: 576–594 (*144–151*); E: 505–514 (*72–82*); the corresponding PDB codes are 3TVJ (MASP-2/SGMI-2), 4DJZ (MASP-1/SGMI-1), 1MD8 (C1r), 1ELV (C1s), 3KCG (fIXa), 1POS (fXa). (**C**) Sequence alignment of human MASPs, C1r, C1s, coagulation fX and fIX including loops 1 and 2. The red color highlights a sequence stretch highly specific of MASP-3. The sequences are listed in a decreasing order of sequence identity of their SP domain compared to MASP-3 (91.1 to 29.7%). The sequences corresponding to MASP-3 in other species are the following: chicken (Q6Q1Q8), mouse (Q8CIR9), xenopus (Q8AXQ8), and amphioxus (Q868H6). (**D**) Zoom on the substrate-binding site, superimposed onto trypsin (dark blue). (**E**) Zoom on the SP loops 1 to 3 of the three MASPs structures in a slightly different orientation. (**F)** Zoom on the fairly similar peptide inhibitor conformations observed for ecotin (red) and SGMI-2 (yellow).

The MASP-3 catalytic triad is defined by H478(*57*), D534(*102*), and S645(*195*) (chymotrypsinogen numbering is indicated in brackets). As a highly conserved binding mode for substrates and inhibitors is observed in serine proteases, the positions of the ecotin residues 78–86 delineate the positions of the common sub-sites (Fig. 6D), as defined by Schechter and Berger [55]. A trypsin-like primary specificity is defined by D639(*189*) at the bottom of the S1 pocket, and by G668(*216*) and G680(*226*) on its edges. The SP loops surrounding the active site, as defined by Perona and Craik [56], modulate the enzyme specificity and efficiency. These loops can be compared to their counterparts in a set of homologous SP domains found in proteases of the complement (MASP-1, MASP-2, C1r, C1s) and coagulation cascades (fIXa, fXa), as illustrated in Figs. 6A and 6B. When MASP-3 is compared to MASP-1 and -2 in complex with the specific SGMI peptide inhibitors [56], the binding similarity extends to two inhibitor loops (Fig. 6A, E, F). MASP-3 also features an extended C-terminal end (Ct on Fig. 6A).

Loops 1–3 finely modulate the enzyme activity and primary specificity. The position of loops 1 and 2 appears to be slightly displaced compared to the other MASPs (Fig. 6E). The sequence of loop 1, including the Y633–Y634(*185–186*) doublet in MASP-3 (Fig. 6E), differs from those in other MASPs, being more similar to coagulation factor IX (Fig. 6C). There is a very short insertion at the beginning of loop 2 in MASP-3 (Fig. 6C, D, E). This insertion is unique among serine proteases, and highly conserved in MASP-3 from different species (Fig. 6C). It occurs just after the conserved critical residue G668(*216*), which corresponds to one of the mutations observed in the 3MC syndrome [18]. P670(*217B*) and the non conserved L642(*192*) form a sort of extra layer on the edge of the S1 sub-site above the conserved C641–C673(*191–220*) disulfide bridge (Fig. 6D, 5E). G668(*216*) and P670(*217B*) also line the S3 sub-site, and, together with Y531(*99*), restrict the space for the P3 substrate residue (Fig. 6D). Possible implications of this unusual insertion in loop 2 will be addressed later in the Discussion. The helix in loop 3 extends up to R618(*173C*) (Fig. 6E). Among the compared SP domains, only C1r exhibits a similar helical extension (Fig. 6B). The S617-R618 residues in loop 3 slightly interact with ecotin S78–S79, thus expanding the substrate-like interactions up to the S6 sub-site (S79 corresponds to P6, Figs. 5D, 6D, 6F).

Compared to other proteases, loops A and D are significantly longer in MASP-3 (Fig. S3) and feature flexible tips. The orientation and position of the beginning of loop A is somehow displaced in MASP-3 (Fig. 6A, B). In this loop, F462(*441*) closes the substrate binding groove at the level of S2’ (Fig. 6D). Loop B adopts an extended conformation in MASP-3 more similar to the longer one of MASP-1 (Fig. 6A). The structure of loop C is also similar in MASP-3 and MASP-1 (Fig. 6A). It should be taken into account that the conformation of several loops, especially B and C, is likely affected by their interaction with ecotin, as will be discussed below.

### Structural Bases of the ecotin/MASP-3 Interaction

In the primary interface, ecotin interacts near the SP domain active site through its flexible loops: 78–86, 48–55, and 98–100 (Fig. 5D). Interactions in this interface are mostly mediated by main-chain/main-chain contacts. These include two canonical central motifs: the main-chain nitrogens of G643(*193*) and S645(*195*), defining the ‘oxyanion hole’, stabilize the ecotin M84 carbonyl; S82 of ecotin is stabilized by two main-chain H-bonds with G668(*216*) of MASP-3. The MASP-3 Y531(*99*) side chain interacts with the ecotin H53-R54 main-chain, as also observed in the case of fXa [52]. The interactions mediated by the ecotin loops 50–53 and 79–86 also share similar features with those mediated by inhibitors of the Pacifastin family, as shown in the case of the SGMI-2/MASP-2 complex (Fig. 6F).

The secondary interface involves contacts between the ecotin loops 33–35, 108–112 and 66–69, and segments 487–489(*60G-60I*), 523–526(*91–94*) and 690–691(*236–237*) of the protease (Fig. 5E). A set of conserved interactions can be delineated in this interface by comparison with other ecotin/protease complex structures. These involve H523(*91*), P524(*92*), F526(*94*), D690(*236*) and W691(*237*) on the protease side, and G66, W67, Y69, R108, N110, L113 on the ecotin side (Fig. 5E). Of note, the mutations Y69F/D70P shown to strongly increase the inhibitory efficiency of ecotin towards trypsin [54] lie in this essential part of the secondary interface. Again, very few protease side-chains (W691(*237*) and H523(*91*)) mediate interactions in this area. Finally, unique additional interactions are provided by the large insertion loop in MASP-3, where T487(*60G*) and V489(*60I*) interact with ecotin S34 and K112, respectively (Fig. 5E).

Following this analysis, we tried to identify the structural bases accounting for the observed differences in ecotin binding by C1r, C1s and the MASPs. The wide MASP-1 substrate-binding site [23], [57] is unlikely to prevent interaction with the flexible ecotin molecule through the primary interface. Ecotin binding through the secondary interface could however be affected by sequence differences at the level of D525(*95*) and N527(*97*) of MASP-3 (Fig. 5E). Bulkier side chains are present at these positions in MASP-1, C1r and C1s, contrary to MASP-2. Moreover, larger insertions in loops B or C, which are clamped in-between two ecotin subunits (Fig. 5C), would strongly restrict their possible conformational space: this applies to the MASP-1 super-extended B loop, and to the larger C1r and C1s C loops (Fig. 6A, B).

### Conformational Flexibility in MASP-3 and its Potential Allosteric Implications

Several surface loops are highly flexible in the MASP-3 SP domain, especially the major insertion loops A and D. Flexibility of some side-chains (mostly charged ones) are observed in loops C and 2, and at the tip of loop 3. Even the N-terminal hydrophobic dipeptide I431(*16*)-I432(*17*) is not as clearly defined as expected in an active serine protease structure. It is stabilized by the activation pocket, which lies in MASP-3 next to the disordered segment 578(*145*)-593(*150*). Very small patches of additional density observed near L441(*26*) further suggest that the N-terminal extremity may adopt alternative orientation(s) up to this level in some molecules of the crystal. Similar features were indeed observed in the structure of complement factor I [58], which, although correctly processed at its activation cleavage site, is in a zymogen-like conformation. No interpretable density was available for the residues of the newly generated N-terminus in most molecules of factor I up to the residue homologous to MASP-3 L441(26). As stated above, the slight N-terminal heterogeneity observed in some batches of the recombinant MASP-3 SP domain could also contribute to this partial local disorder.

**Figure 7:**
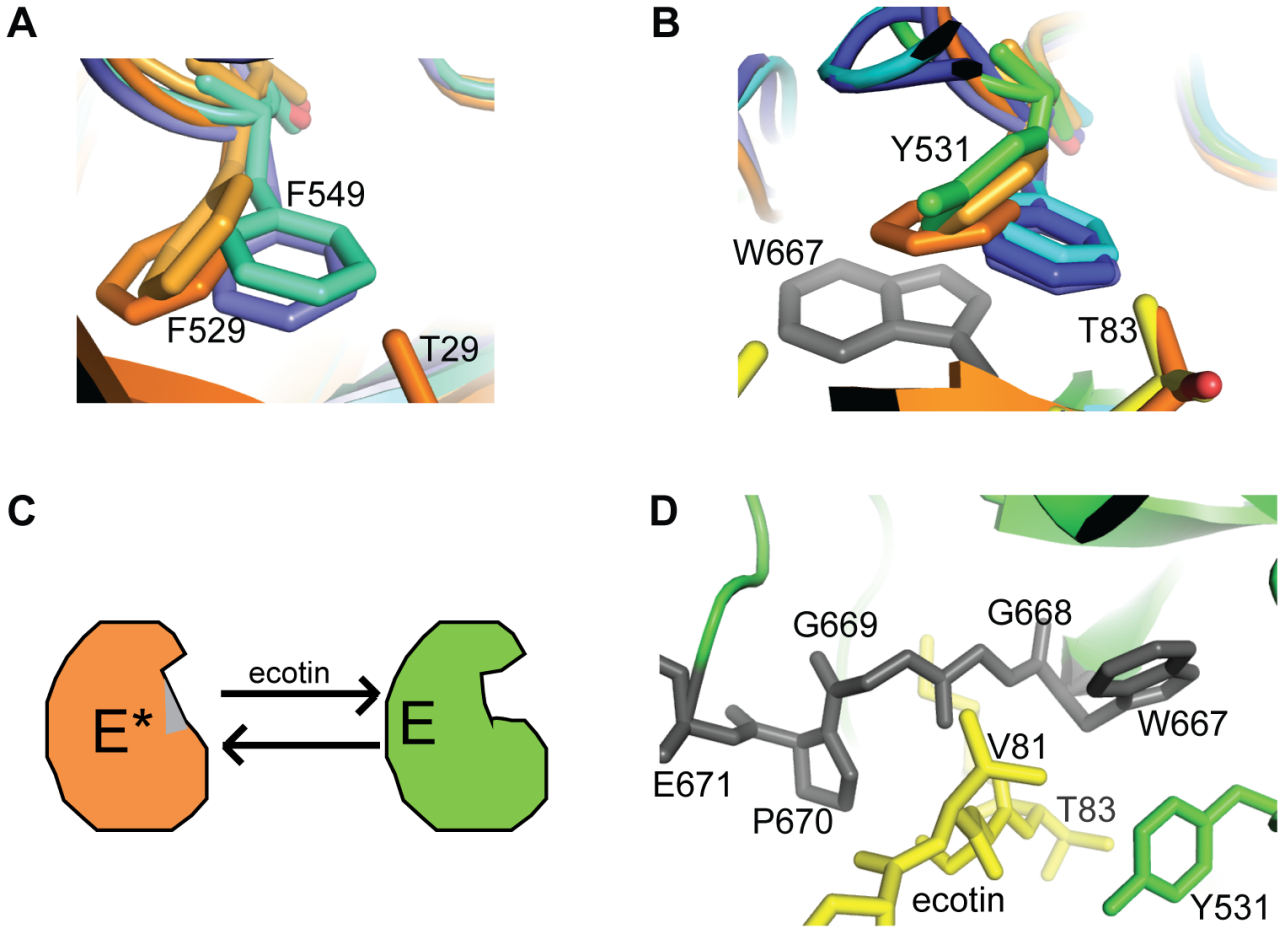
Ecotin-induced displacement and proposed conformational equilibrium of MASP-3. (**A**) Displacements of the homologous Phe549 and Phe529 in MASP-1 and MASP-2 (in cyan and blue for the free enzyme, respectively) upon SGMI inhibitor binding (left in orange). (**B**) The position of the homologous Tyr531 in MASP-3 (green) clusters with the ‘displaced’ positions and not with the ‘free’ positions of MASP-1 and MASP-2. The homologous side-chain in free C1r (dark blue) perfectly fits between those of free MASP-1 and MASP-2. It is also the case for C1s (not shown). (**C**) Scheme illustrating the proposed conformational equilibrium between an inactive E* conformation and the active E conformation observed upon interaction with ecotin. The segment *215–217,* which collapses in the substrate-binding site in the E* or Z* states is shown in grey. (**D**) Position of the segment 667–671 (in grey) homologous to *215–217*, in close proximity to Tyr531 and the substrate binding site (see ecotin in yellow). This segment includes the specific MASP-3 insertion in loop-2 illustrated in Fig. 6.

Another indirect mark of flexibility resides in the probable ecotin-constrained local conformation(s) of some surface loops. Since the crystal structure of MASP-3 alone is not available, the free and ecotin-bound conformations cannot be compared. However, ecotin-induced conformational changes have been observed previously in the case of thrombin and factor Xa [52], and significant conformational changes were also observed in the catalytic fragment of MASP-2 in complex with SGMI-2 [57]. Evidence of such a change may be inferred from the comparison of the position and orientation of Tyr531(99) in MASP-3 and its homologues. In MASP-1 and -2, the positions of the corresponding Phe549 and Phe529 side chains, respectively, are significantly displaced by their interaction with the peptide inhibitor (Fig. 7A). In fXa, this residue is significantly displaced by its close interaction with loop 50 of ecotin [52]. Its conformation in ecotin-bound MASP-3 is far more similar to that in the ecotin-bound fXa (not shown) or in the SGMI-bound MASP-1 and -2 than in the free forms of MASP-1, MASP-2, C1r, C1s and fXa (Fig. 7B). As loops B and C are clamped in-between two ecotin molecules (Fig. 5C), they likely require an adaptive conformational change in MASP-3 to accommodate ecotin binding, which would be consistent with the lower association rate observed (Table 3).

### Towards a Plausible Conformational Switch in the MASP-3 SP Domain

Such a conformational flexibility could offer a clue to resolve the following apparent MASP-3 paradox: on the one hand, this protease clearly exhibits poor enzymatic properties *in vitro*; on the other hand, its structure in complex with ecotin is quite similar to that of ‘classical’ active proteases. In particular, this structure does not explain why C1-inhibitor, the physiological inhibitor of all other proteases of the C1r/C1s/MASP family, does not bind to MASP-3. The concept of allostery was recently introduced as a general property of the trypsin-like protease fold [27], [59], [60]. Besides the activation cleavage transforming a zymogen (Z) into an enzyme (E), this concept adds a possible conformational equilibrium between an active E (or Z) conformation and an inactive one (E* or Z*). Access to the binding site is blocked in E* or Z*, but a conformational switch to the active form can be provided *in situ* by an activator and/or a substrate. Our observations would thus be consistent with the hypothesis that the MASP-3 SP domain is seen in the E state in the crystal structure, as a result of its interaction with ecotin, whereas it is mainly stabilized in the E* state when its catalytic activity is assayed *in vitro* (Fig. 7C). Two major arguments give credit to this hypothesis: (i) the poor activity of free thrombin (in the E* state) has been correlated to its conformational equilibrium [61], [62] and MASP-3 also exhibits significant conformational flexibility in its SP domain, even in the crystal structure; (ii) the MASP-3 specific insertion in loop 2 occurs in the 667–671(*215–218*) segment (in grey in Fig. 7D), homologous to the segment collapsing into the substrate-binding site in the E* and Z* structures, thus blocking its access [27], [60]. This insertion could thus promote the collapse of this fragment 667–671(*215–218*) into the substrate-binding site in the absence of ecotin.

How could ecotin bind to MASP-3 in its initial E* state? A likely hypothesis is that ecotin first binds through the secondary interface, thereby triggering a conformational switch opening the substrate binding site, which allows in turn the second ecotin molecule to bind through the primary interface. The arguments supporting this hypothesis are the following: (i) ecotin is unlikely to bind first through the primary interface since the interactions observed at this interface are quite poor, implying very few side-chains; of note, the P1 residue in ecotin is Met84 instead of the preferred lysine or arginine side-chains. The fact that ecotin does not bind MASP-1 is likely due to steric conflicts at the secondary interface and not at the primary interface, as discussed previously. (ii) ecotin has been shown to trigger such a conformational switch by binding to the exosite 2 of thrombin via its secondary interface [50], [56], and we have delineated a set of similar interactions in the secondary interface of the ecotin/MASP-3 complex; (iii) the reduced association rate constant observed in the case of MASP-3 (Table 3) would be fully consistent with the requirement of conformational changes prior to binding through the primary interface.

### Conclusion

This study describes a further example of the wide inhibitory spectrum of ecotin and illustrates how this inhibitor makes use of two interfaces targeting essential main-chain functional elements of trypsin-like proteases. In particular, conserved interactions within the secondary interface are delineated here for the first time. Our experimental data together with recent analyses of trypsin-like proteases support the hypothesis that, in order to become fully ‘active’, the SP domain of MASP-3 not only requires an activation cleavage but also needs a conformational switch triggered by a cofactor and/or a substrate. The occurrence of such a conformational equilibrium featuring a blocked E* state would explain why C1-inhibitor does not bind MASP-3, and why the activity of this protease has proven difficult to study up to now *in vitro*. Such a conformational switch will require interactions at exosites probably located in the vicinity of the 487–489, 523–526 and 690–691 segments of MASP-3, involved in ecotin binding through the secondary interface. Such exosites would significantly differ, in terms of function and location, from the exosites mediating C4 interaction in MASP-2 [63].

A major implication of this hypothesis is that the function of MASP-3 would be tightly regulated, as it is the case for biologically crucial enzymes such as thrombin or complement factor D and I [27], [58]. Tight control of MASP-3 activity by a substrate or a cofactor will ensure a high local MASP-3 concentration relative to the substrate, which would be consistent with the observations that MASP-3 proteolytic activity requires high enzyme concentrations relative to the substrate. This would be consistent with the important biological role of MASP-3 *in vivo*, as revealed recently by the discovery that several of its mutations are associated with the 3MC syndrome. The residues associated with the disease include the catalytic His 478, the essential Gly 668 and other residues close to the primary interface ([17], [18], Fig. 5C), thus strongly suggesting that the 3MC syndrome is a consequence of the loss of enzymatic activity of MASP-3. One of the proposed MASP-3 substrates, IGFBP-5, is involved in developmental processes, as is also CL-11, as shown by studies in zebrafish embryos [17].

This structure opens the way to protein engineering to obtain more active variants to further study MASP-3 *in vitro*. Specific inhibitors could be designed in light of the structure described here in complex with ecotin. This could help in the future to better elucidate the possible role of MASP-3 in complement activation or its other important functions.

## Supporting Information

Figure S1 Example of the quality of the map in the area discussed in the text. MASP-3 residues are in green, ecotin in red. This section shows the main part of the segment 667–670 (in grey in Fig. 7D), including the G669-P670 insertion in loop 2 (Fig. 6C). The sidechain of Tyr531 (Fig. 7B) is also displayed. This 2mFo-dFc map section is countered at a 1 sigma level. Coot (Emsley, Lohkamp and Cowtan, 2010, Acta Cryst. D66, 486–501) and its screenshot option was used to generate this image.
(PDF)

Figure S2 SDS-PAGE analysis of the cleavage of IGFBP5 by the MASP-3 SP domain. IGFBP5 (2.8 µg, 100 pmols) was incubated for 16 h at 37°C either alone (lane 3) or in the presence of 0.7 µg (20 pmols) of MASP-3 SP (lane 4) or 1.6 µg (20 pmols) of C1s (lane 1). Lane 5: MASP-3 SP alone (0.7 µg). The molecular masses of reduced standard proteins are indicated on the right side.
(PDF)

Figure S3 Multiple sequence alignments of a set of complement and coagulation pro-teases serine protease domains. It includes the complement C1r, C1s, MASP-1 and MASP-2 proteases, coagulation factors 9, 10, 11 and thrombin; Rat trypsin as a reference; MASP-3 from human and other species. The surface loops defining the substrate specificity and catalytic efficiency are noted on the top of the align-ment, following the nomenclature introduced by Perona and Craik (1997, JBC, 272, 29987-90). Yellow diamonds locate the position of the human MASP-3 glycosylation sites.
(PDF)
