# Supplementary material for: The Serine Protease Domain of MASP-3: Enzymatic Properties and Crystal Structure in Complex with Ecotin
Source: PLoS One. 2013 Jul 4;8(7):e67962. doi: 10.1371/journal.pone.0067962 (PMC3701661; doi:10.1371/journal.pone.0067962)

Figure S1. Example of the quality of the map in the area discussed in the text. MASP-3 residues are in green, ecotin in red. This section shows the main part of the segment 667-670 (in grey in Fig. 7D), including the G669-P670 insertion in loop 2 (Fig. 6C).The sidechain of Tyr531 (Fig. 7B) is also displayed. This 2mFo-dFc map section is countered at a 1 sigma level. Coot (Emsley, Lohkamp and Cowtan, 2010, Acta Cryst. D66, 486–501) and its screenshot option was used to generate this image.

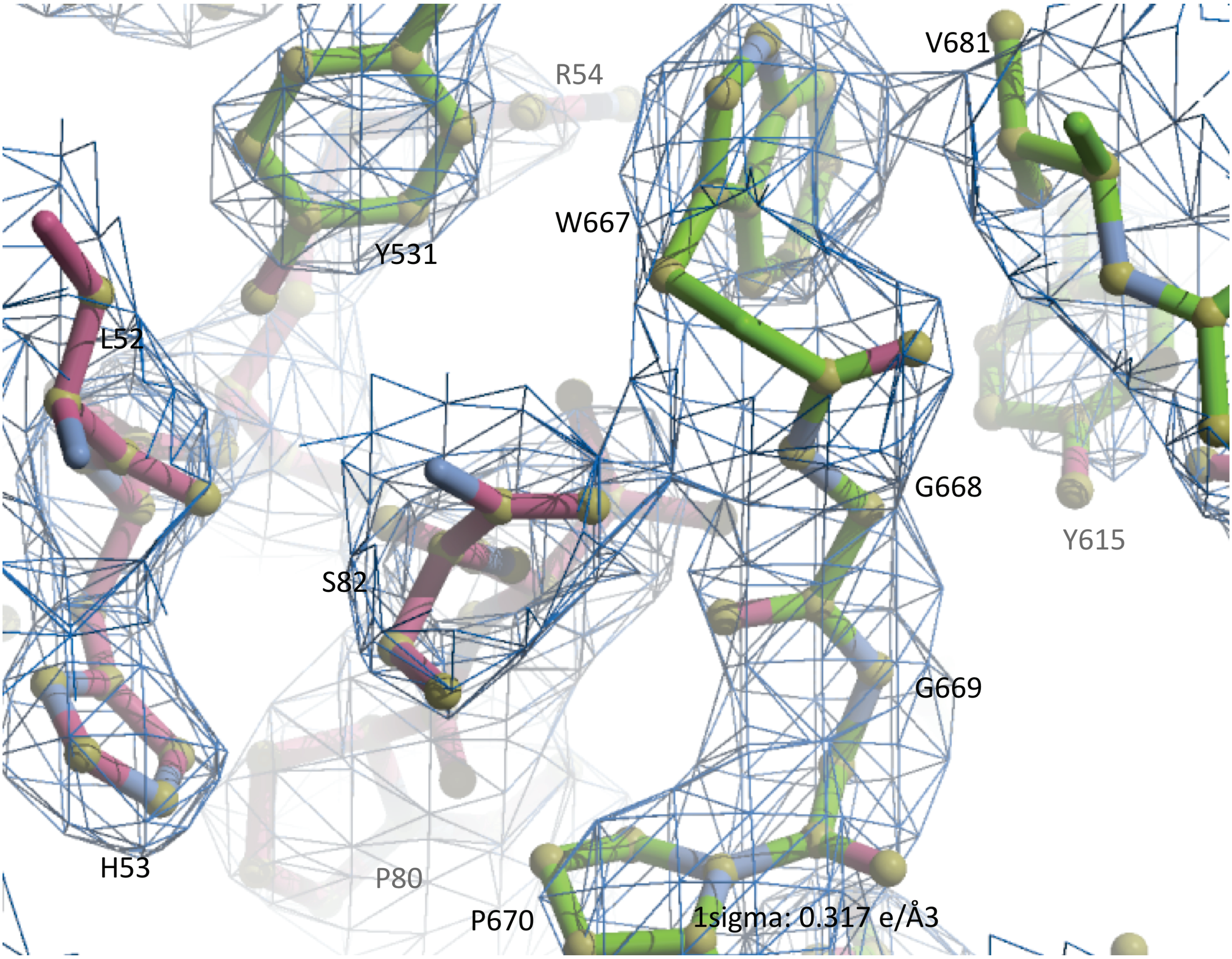

Supplement: Figure S1 — Example of the quality of the map in the area discussed in the text. MASP-3 residues are in green, ecotin in red. This section shows the main part of the segment 667–670 (in grey in Fig. 7D), including the G669-P670 insertion in loop 2 (Fig. 6C). The sidechain of Tyr531 (Fig. 7B) is also displayed. This 2mFo-dFc map section is countered at a 1 sigma level. Coot (Emsley, Lohkamp and Cowtan, 2010, Acta Cryst. D66, 486–501) and its screenshot option was used to generate this image. (PDF) [file pone.0067962.s001.pdf]
