# Supplementary material for: The Serine Protease Domain of MASP-3: Enzymatic Properties and Crystal Structure in Complex with Ecotin
Source: PLoS One. 2013 Jul 4;8(7):e67962. doi: 10.1371/journal.pone.0067962 (PMC3701661; doi:10.1371/journal.pone.0067962)

Figure S2. SDS-PAGE analysis of the cleavage of IGFBP5 by the MASP-3 SP domain. IGFBP5 (2.8 µg, 100 pmols) was incubated for 16 h at 37°C either alone (lane 3) or in the presence of 0.7 µg (20 pmols) of MASP-3 SP (lane 4) or 1.6 µg (20 pmols) of C1s (lane 1). Lane 5: MASP-3 SP alone (0.7 µg). The molecular masses of reduced standard proteins are indicated on the right side.

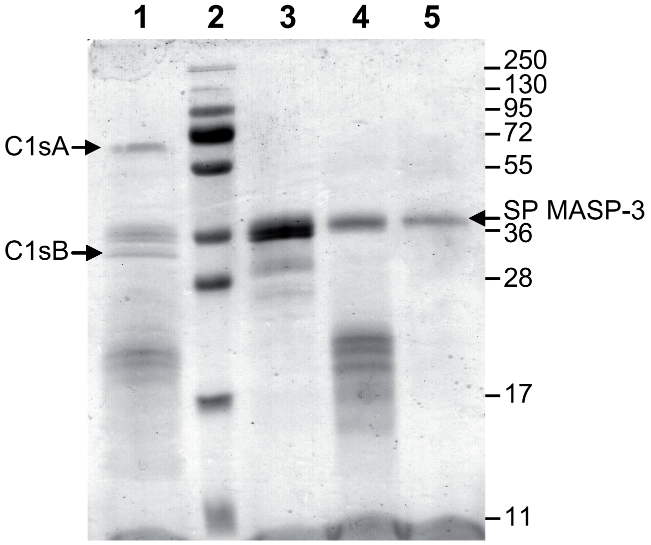

Supplement: Figure S2 — SDS-PAGE analysis of the cleavage of IGFBP5 by the MASP-3 SP domain. IGFBP5 (2.8 µg, 100 pmols) was incubated for 16 h at 37°C either alone (lane 3) or in the presence of 0.7 µg (20 pmols) of MASP-3 SP (lane 4) or 1.6 µg (20 pmols) of C1s (lane 1). Lane 5: MASP-3 SP alone (0.7 µg). The molecular masses of reduced standard proteins are indicated on the right side. (PDF) [file pone.0067962.s002.pdf]
