# Supplementary material for: The Serine Protease Domain of MASP-3: Enzymatic Properties and Crystal Structure in Complex with Ecotin
Source: PLoS One. 2013 Jul 4;8(7):e67962. doi: 10.1371/journal.pone.0067962 (PMC3701661; doi:10.1371/journal.pone.0067962)

Figure S3. Multiple sequence alignments of a set of complement and coagulation proteases serine protease domains. It includes the complement C1r, C1s, MASP-1 and MASP-2 proteases, coagulation factors 9, 10, 11 and thrombin; Rat trypsin as a reference; MASP-3 from human and other species. The surface loops defining the substrate specificity and catalytic efficiency are noted on the top of the alignment, following the nomenclature introduced by Perona and Craik (1997, JBC, 272, 29987-90). Yellow diamonds locate the position of the human MASP-3 glycosylation sites.

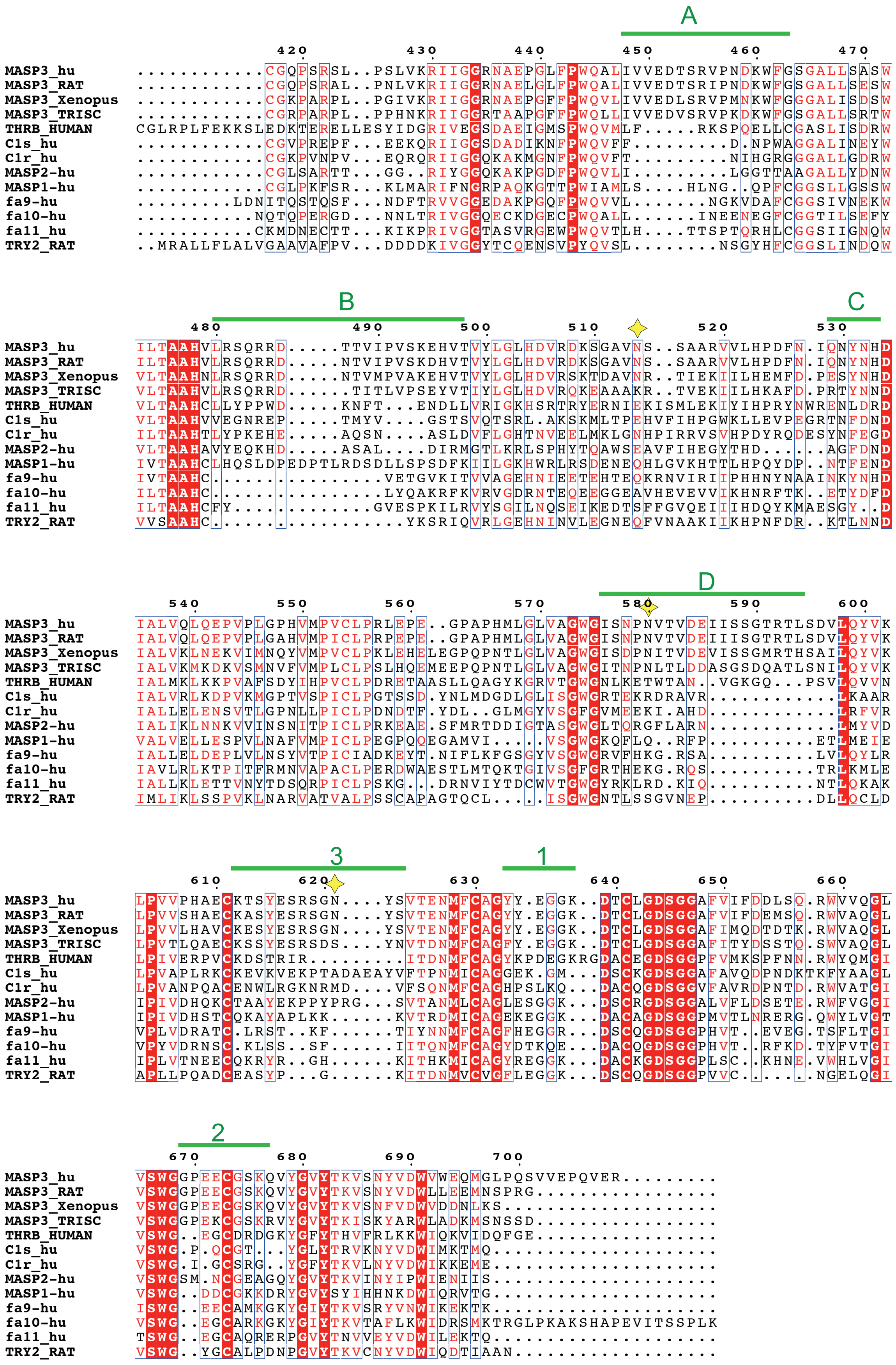

Supplement: Figure S3 — Multiple sequence alignments of a set of complement and coagulation pro-teases serine protease domains. It includes the complement C1r, C1s, MASP-1 and MASP-2 proteases, coagulation factors 9, 10, 11 and thrombin; Rat trypsin as a reference; MASP-3 from human and other species. The surface loops defining the substrate specificity and catalytic efficiency are noted on the top of the align-ment, following the nomenclature introduced by Perona and Craik (1997, JBC, 272, 29987-90). Yellow diamonds locate the position of the human MASP-3 glycosylation sites. (PDF) [file pone.0067962.s003.pdf]
